# Synthesis of Gold Nanoparticles over CoAl Mixed Oxide for Ethanol Oxidation Reaction

**DOI:** 10.3390/molecules29102285

**Published:** 2024-05-12

**Authors:** Guillaume Rochard, Eric Genty, Jean-Marc Giraudon, Christophe Poupin, Jean-François Lamonier, Stéphane Siffert, Valeria La Parola, Leonarda Francesca Liotta, Renaud Cousin

**Affiliations:** 1 Unité de Chimie Environnementale et Interactions sur le Vivant (UCEiV), Université du Littoral Côte d’Opale (ULCO), 59140 Dunkerque, France; grochard.guillaume@gmail.com (G.R.); eric.genty@univ-littoral.fr (E.G.); christophe.poupin@univ-littoral.fr (C.P.); stephane.siffert@univ-littoral.fr (S.S.); 2 Unité de Catalyse et Chimie du Solide (UCCS), UMR 8181, Université de Lille, CNRS, Centrale Lille, Université Artois, 59000 Lille, Francejean-francois.lamonier@univ-lille.fr (J.-F.L.); 3 Institute for the Study of Nanostructured Materials (ISMN)-CNR, Via Ugo La Malfa, 153, 90146 Palermo, Italy; valeria.laparola@cnr.it

**Keywords:** gold nanoparticles, DP method, CoAl mixed oxides, LDH, ethanol total oxidation

## Abstract

Catalytic total oxidation is an effective technique for the treatment of industrial VOCs principally resulting from industrial processes using solvents and usually containing mono-aromatics (BTEX) and oxygenated compounds (acetone, ethanol, butanone). The aim of this work is to deposit gold nanoparticles on CoAl mixed oxide issued from layered double hydroxide (LDH) precursor by using the deposition precipitation (DP) method, which is applied with two modifications, labeled method (A) and method (B), in order to enhance the interaction of the HAuCl_4_ precursor with the support. Method (A) involves the hydrolysis of the HAuCl_4_ precursor after addition of the support, while in method (B), the gold precursor is hydrolyzed before adding the support. The two methods were applied using as support the CoAl mixed oxide and the LDH precursor. Samples were characterized by several physical chemical techniques and evaluated for ethanol total oxidation. Method (B) allowed the ethanol oxidation activity to be enhanced for the resulting Au/CoAlOx catalysts thanks to the high surface concentration of Co^2+^ and improved reducibility at low temperature. The presence of gold permits to minimize the formation of by-products, notably, methanol, allowed for a total oxidation of ethanol at lower temperature than the corresponding support.

## 1. Introduction

Volatile organic compounds (VOCs) are known as one of major contributors to atmospheric pollution. Their anthropic release is particularly significant in industrialized areas and has an impact on health, environment, and construction materials. The majority of these emissions concern BTEX compounds (benzene, toluene, and xylenes) and oxygenated compounds like ethanol, methanol, and acetone [1,2,3].

An efficient method for reducing VOC emissions is catalytic total oxidation into carbon dioxide and water. Catalytic total oxidation is an economical alternative to the use of thermal oxidation due to the obtention of total oxidation of VOCs (150–300 °C for catalytic oxidation compared to 600–800 °C in the case of thermal oxidation). Moreover, catalytic oxidation allows better selectivity to be obtained for the desired compounds (CO_2_ and water for total oxidation) by limiting the formation of by-products that may be more toxic than the initial molecules. The catalytic material used for the total oxidation of VOCs in industrial application is frequently a noble metal-based catalyst (with Pd and Pt) [4,5,6,7]. During the past decades, many studies have established that gold nanoparticles on a reducible support have remarkable catalytic activity for CO or oxygenated VOCS total oxidation [8,9,10,11]. In order to obtain reducible support, one interesting way is to use layered double hydroxide (LDH). Previous studies have shown that the replacement of Mg^2+^ (present in natural hydrotalcite) by other divalent cations (Co^2+^, Cu^2+^, Mn^2+^, etc.) allows for interesting performance for VOC oxidation to be obtained [4,12,13,14]. This interesting performance has been explained by the high reducibility at low temperature and the good dispersion of the oxide phase principally. Moreover, the mixed oxides issued from this synthesis method present good thermal stability and resistance to poisoning. The combination of gold nanoparticles on mixed oxides issued from the LDH route have already shown interesting performance for CO and VOC oxidation [15,16,17]. However, the catalytic activity of Au-based mixed oxides is dependent on the synthesis method for the deposition of gold nanoparticles due to the influence of the reactivity with nanoparticle size [11,18,19,20]. The procedure principally used in the literature is the deposition–precipitation method. This process has already shown interesting results compared to the impregnation process, thanks to the lower gold nanoparticle size attained during controlled deposition–precipitation steps. The size of gold nanoparticles depends on various parameters, like the type of support (reducible or not reducible oxide, point of zero charge), precipitating agent (urea, NaOH, etc.), and pH value [21]. In addition, the deposition–precipitation method is mainly suitable for highly reducible support oxides, such as CeO_2_, CeO_2_-TiO_2_, or CeO_2_-ZrO_2_, which can stabilize gold through a metal–support interaction [22].

Previous studies in our laboratory have shown that Co-Al mixed oxide catalysts present interesting catalytic activity for the total oxidation of toluene [4,12,13,14]. Therefore, Co-Al mixed oxide synthetized by layered double hydroxides was considered an eligible support for gold nanoparticle deposition. In the present study, Au catalysts were prepared by the deposition–precipitation (DP) method over CoAl mixed oxides to be investigated for the ethanol total oxidation reaction. A first set of catalysts was prepared by performing the hydrolysis of the HAuCl_4_ precursor after mixing with the CoAl oxide. After some observations discussed in this paper, the procedure of the deposition of gold was performed on the HDL precursor used as the support, and a modification of the procedure wherein the gold precursor was pre-hydrolyzed before the deposition, called method (B), was applied. The influence of deposited gold content (0.5–1.5 wt%) on the ethanol oxidation activity was evaluated. Several physicochemical characterizations were performed to understand the influence of the methodology on the catalytic activity and selectivity to secondary oxidation products.

## 2. Results

**Figure 1:**
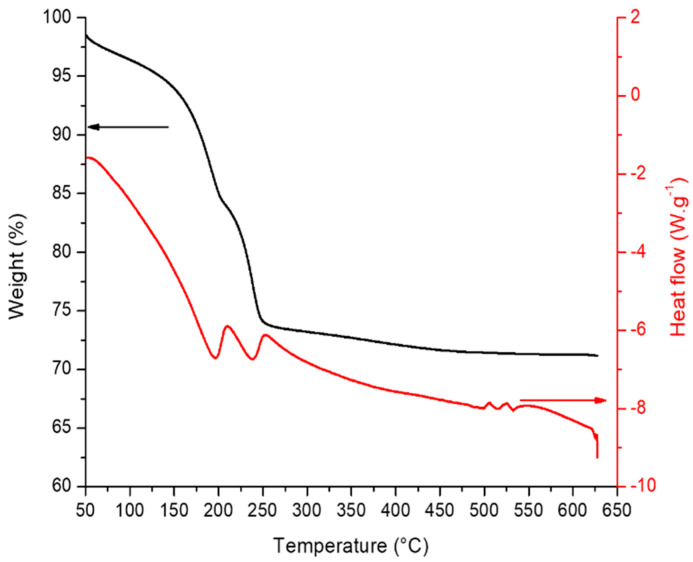
Weight loss and heat flow vs. temperature for CoAl HT material.

The layered double hydroxide (LDH) structure was used as a precursor to the Co-Al mixed oxide obtained after the calcination step. With the aim of evaluating the lowest temperature for the destruction of the LDH structure and the formation of the mixed oxide, DTA/TG analysis was performed. The DTA/TG profile for the CoAlHT compound is reported in Figure 1. The main weight loss (28%) occurring in the range of 50–250 °C was accompanied by an endothermic peak corresponding to the removal of weakly bonded water in the interlayer space of the hydrotalcite up to 250 °C and an overlap with an exothermic peak corresponding to nitrate–carbonate decomposition above 200 °C [23]. By further increasing the temperature, a slight mass decrease still occurred up to a stable value at 500–550 °C. On this basis, two temperatures were selected for calcining the HT precursor: 250 °C in order to obtain the mixed oxide, labelled CoAlOx 250, and 500 °C in order to obtain a reference material, labelled CoAlOx 500, according to our previous investigations [4,13]. After gold deposition, all the catalysts were calcined at the same temperature, 250 °C.

**Figure 2:**
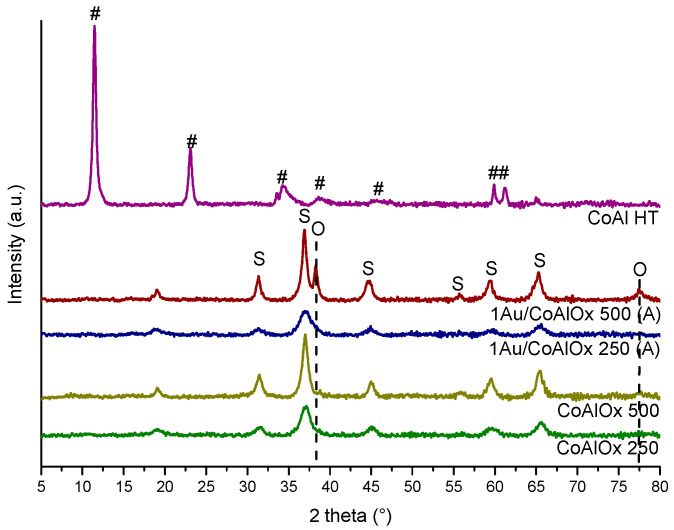
XRD pattern of CoAl HT, CoAl Ox supports, and Au deposited on mixed oxides (by DP method, method (A)) (#: Co_6_Al_2_(OH)_16_CO_3_, 4 H_2_O; S: Co_3_O_4_, CoAl_2_O_4_, and Co_2_AlO_4_; O: Au^0^).

X-ray diffraction patterns of LDH and CoAl Ox obtained after calcination at 250 °C and at 500 °C, respectively, are displayed in Figure 2. For the CoAl HT material, diffraction peaks at 2θ = 11.5°, 23.1°, 34.6°, 38.4°, 59.9°, 61.2°, and 65° were representative of hydrotalcite-like materials (JCPDF: 51-0045). After calcination at 250 °C, a phase modification was observed. The 2θ peaks at 19.0°, 31.3°, 38.5°, 44.8°, 59.4°, and 65.2° corresponded to Co_3_O_4_ (JCPDF: 42-1467), CoAl_2_O_4_ (JCPDF: 44-0160), and Co_2_AlO_4_ (JCPDF: 38-0814) spinel oxide phases. The differentiation of these three phases was complicated due to the fact that characteristic diffraction peaks are close in intensity and position [23,24]. This XRD pattern confirms that after calcination at 250 °C the formation of the mixed oxide took place; however, lower crystallinity was observed for the spinel phase with respect to the sample calcined at 500 °C.

Deposition of gold, 1 wt%, was performed using the deposition–precipitation method (DP method) (see Section 3.1) on two different portions of mixed oxide obtained after calcination at 250 °C and 500 °C, respectively. The resulting catalysts were labelled 1Au/CoAl Ox 500 (A) and 1Au/CoAl Ox 250 (A).

X-ray diffraction patterns of the Au-supported catalysts are plotted in Figure 2 and were compared with the corresponding Co-Al mixed oxides. For gold-based samples, spinel oxide phases were observed with an intensity similar to that of the supports. Features typical of gold metallic phase were visible at ~38 and 78° 2θ for the 1Au/CoAl Ox 500 catalyst (JCPDF: 04-0784). An evaluation of Au particle size was performed. For the 1Au/CoAl Ox 500 sample (A), the particle size was estimated at 14 nm, while for the 1Au/CoAl Ox 250 sample (A) it was complicated due to the low intensity of the peak. The other two peaks characteristic of Au^0^ at 44.4° and 64.6° were covered by the signals of spinel oxide phases.

Conversely, well-dispersed gold nanoparticles were formed in the 1Au/CoAl Ox 250 (A) catalyst, showing only a barely visible shoulder at ~38°, corresponding to the main reflection (111) of metallic Au. Moreover, with the peaks of the spinel oxide phases detected for 1Au/CoAl Ox 250 being larger than those of the corresponding support CoAl Ox 250, we cannot exclude that some Au ionic species may have been stabilized into the lattice of the support oxide, perturbing its crystallinity. By comparing the Au features detected in the two gold catalysts discussed above, it can be concluded that the different calcination temperatures of the support at 500 °C instead of 250 °C inducing a drastic dihydroxylation of the surface along with a dramatic decrease in specific surface area (113 vs. 275 m^2^·g^−1^) and pore volume (0.7 vs. 0.95) were responsible for the poor anchoring of the [AuCl_4-x_OH_x_]^−^ species during the DP step and for the sintering of some Au particles during calcination [18].

**Figure 3:**
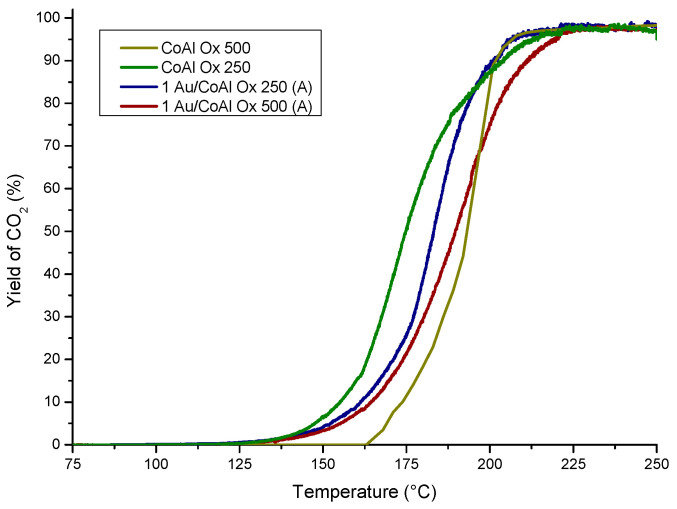
CO_2_ production during the ethanol conversion vs. temperature over 1 wt% Au catalysts deposited by method (A) over CoAl Ox 500 and CoAl Ox 250.

The mixed oxides and gold-based catalysts were tested for total oxidation of ethanol into carbon dioxide and water. Some by-products were also observed, and a discussion about these by-products will be carried out in this paper. The yield of CO_2_ during the ethanol oxidation are represented in Figure 3.

The catalytic order based on T_50_ (temperature corresponding to 50% of conversion of ethanol into CO_2_) followed this order: CoAl Ox 250 (175 °C) ≥ 1Au/CoAl Ox 250 (A) (184 °C) > 1Au/CoAl Ox 500 (A) (189 °C) > CoAl Ox 500 (194 °C).

Based on this result, an influence of the calcination temperature was observed for the mixed oxides, with better performance for the sample calcined at a lower temperature. This difference can be justified by the higher specific surface area (275 m^2^·g^−1^ for CoAl Ox 250 vs. 113 m^2^·g^−1^) for the sample calcined at 500 °C. The addition of gold to the CoAl Ox 250 support did not improve the catalytic activity, especially at low temperature, where the catalyst 1Au/CoAl Ox 250 (A) was barely active. Such a finding, apparently in contradiction with the presence of well-dispersed gold, as argued on the basis of XRD analysis (Figure 2), can be explained by the strong decrease in surface area and porosity (155 m^2^·g^−1^ vs. 275 m^2^·g^−1^) of the catalyst with respect to CoAl Ox 250. Such a phenomenon likely induced inward diffusion of Au nanoparticles inside the support. Conversely, the expected positive catalytic effect of gold was confirmed at low temperature in the 1Au/CoAl Ox 500 (A), with a similar specific surface as the support, although with the presence of relatively big Au particles (Figure 2).

Based on the low enhancement in catalytic performance for the total oxidation of ethanol, we decided to use the LDH precursor as a support instead of the CoAl mixed oxide. The support and the samples containing gold were calcined only one time at 250 °C after gold deposition in the case of gold deposition. Indeed, the previous results show that the sample calcined at 250 °C presented the best ethanol conversion at low temperature compared to the samples calcined at 500 °C. During the first synthesis, using method (A) and the LDH precursor, a darkening of the solution was observed as soon as the suspension of the support was in contact with the HAuCl_4_ precursor, suggesting a reduction in gold and the formation of gold particles not interacting with the support. In order to control this phenomenon of a reduction in gold not in interaction with the support, a second method, method (B), was used. For this method, the classical procedure was modified and the pH of an appropriate volume of HAuCl_4_·3H_2_O was fixed at 8 by adding K_2_CO_3_ (0.05 mol L^−1^), allowing for hydrolysis of the gold precursor that easily precipitated on the CoAl HT that was added at the end. By following this procedure, three Au catalysts were prepared and calcined at 250 °C, which were labelled yAu/CoAl HT (B), with y = 0.5, 1, and 1.5 wt%.

For comparison purposes and to ascertain whether the darkening of the solution still occurred when the suspension of the support was in contact with the HAuCl4 precursor, even in the presence of CoAl HT, thereby hindering the stabilization of [AuCl_4-x_(OH)_x_]^−^ species within the layered structure, Au 1 wt% was deposited using method (A) over CoAl HT. The resulting catalyst was named 1Au/CoAl HT (A).

**Figure 4:**
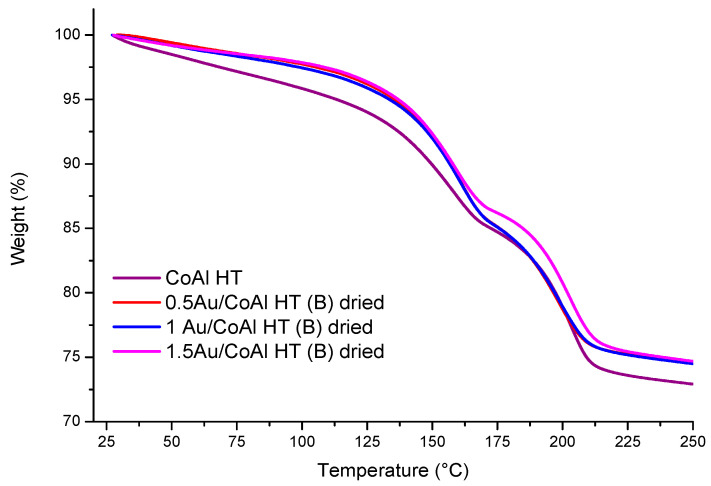
TGA profiles for CoAl HT and dried Au/CoAl HT (B) samples.

In order to investigate the possible interaction between the CoAl HT and the [AuOH_4_]^−^ species formed at pH 8 by hydrolysis of the HAuCl_4_ precursor, TG analyses were carried out on the dried materials recovered after gold deposition–precipitation by using method (B). The weight loss curves vs. temperature for dried catalysts Au/CoAl HT with three gold loadings (0.5Au/CoAl HT, 1Au/CoAl HT, and 1.5Au/CoAl HT) are displayed in Figure 4.

First of all, it was observed that the deposition of gold influenced the thermal behavior of the CoAl HT. Indeed, the gold loading affected the temperature of the thermal decomposition of the hydrotalcite, which occurred at a higher temperature with respect to the Au-free material and with a slight decrease in weight loss. This difference could be explained by the effect of [CO_3_]^2−^ substitution by the [Au(OH)_4_]^−^ species present on the interlayer. Therefore, it can be deduced that the deposition–precipitation of Au species was effective in the CO_3_^2−^ substitution, implying a stabilization of [Au(OH)_4_]^−^ species in the layered structure. This deposition on the interlayer induced better dispersion of gold species compared to the previously discussed 1Au/CoAl Ox 500 and 1Au/CoAl Ox 250 synthetized by method (A).

**Figure 5:**
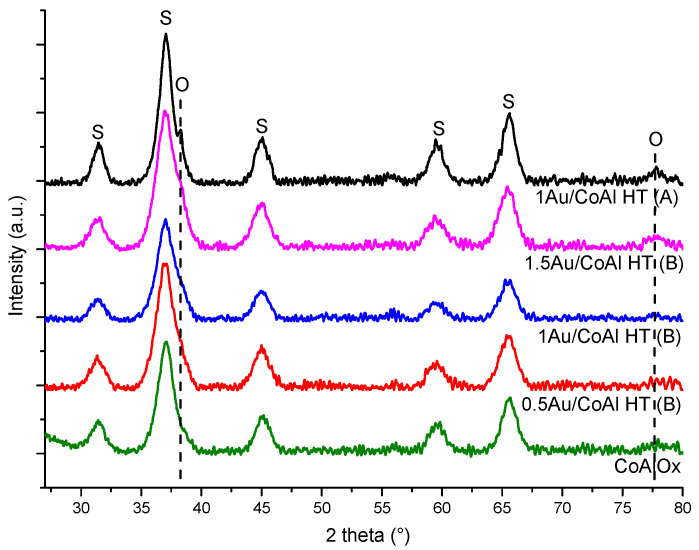
XRD patterns of CoAl Ox 250 support and gold deposited over CoAl HT with the different preparation methods (S: Co_3_O_4_, CoAl_2_O_4_, and Co_2_AlO_4_; O: Au^0^).

The X-ray diffraction patterns of the supported Au catalysts, calcined at 250 °C, are compared in Figure 5.

All gold-based catalysts (Figure 5) presented spinel phases (Co_3_O_4_ (JCPDF: 42-1467), CoAl_2_O_4_ (JCPDF: 44-0160), and Co_2_AlO_4_ (JCPDF: 38-0814)) with similar intensity and FWHM as the parent support (CoAl Ox 250). Concerning the gold metallic phase (Au^0^, JCPDF: 04-0784), features at ~38 and 78° were observed only for the solid 1Au/CoAl HT (A) [11,25]. Conversely, in the catalysts synthesized using method (B), clear signals of metallic Au were not visible, with the exception of a shoulder around ~38° in the peak of the spinel phase. This observation suggests a high dispersion of gold nanoparticles achieved through method (B), indicating a potential substitution of CO_3_^2−^ by [Au(OH)_4_]^−^ species within the interlayer of the CoAl HT material.

**Figure 6:**
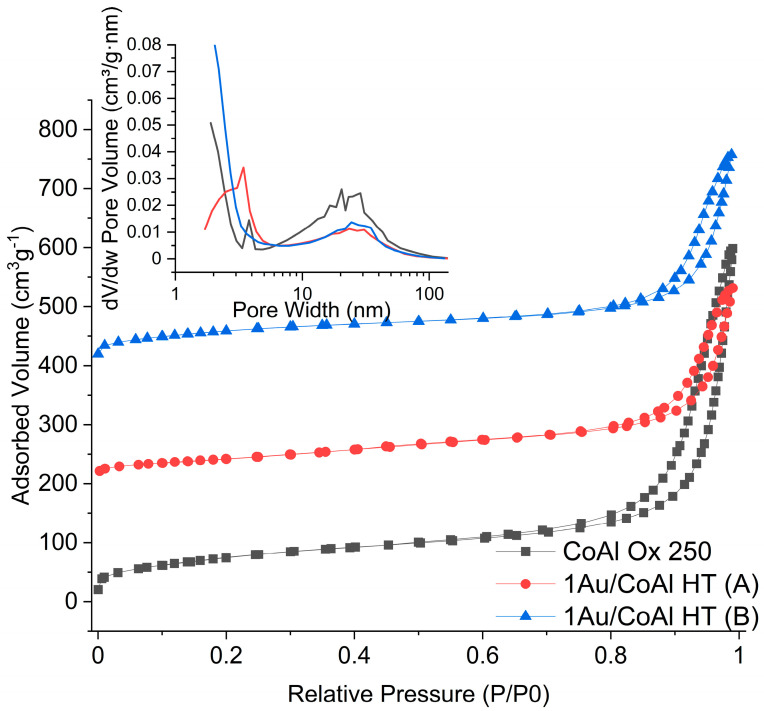
Adsorption–desorption isotherm for CoAl Ox 250, 1Au/CoAl HT (A), and 1Au/CoAl HT (B). In the inset is the corresponding pore size distribution.

The textural properties for all catalytic materials are reported in Table 1, and the adsorption–desorption isotherm along with the pore size distribution is shown in Figure 6 for selected catalysts. The CoAl Ox 500 and the corresponding catalyst 1Au/CoAl Ox 500 (A) exhibited similar specific surface area but with a lower pore volume and mean pore size in the case of the catalyst, in accordance with the formation of big Au particles (around 14 nm), as detected by XRD (Figure 2). All the gold catalysts prepared on the LDH precursor suffered a decrease in surface area, pore size, and pore volume with respect to the support calcined at the same temperature, CoAl Ox 250, and the effect was dependent on method (A) or (B). Both samples 1Au/CoAl Ox 250 and 1Au/CoAl HT prepared by method (A) showed the lowest surface and pore volume, confirming a detrimental effect of the formation of metallic gold particles during the DP step (darkening of the solution). The catalysts prepared by method (B) somehow preserved the morphological properties typical of CoAl Ox 250, although pore size and volume were affected by Au loading. This fact could be indicative of the already mentioned intercalation of the formation of [Au(OH)_4_]^−^ in hydrotalcite structures, which gives rise to a more compact morphology after calcination.

**Figure 7:**
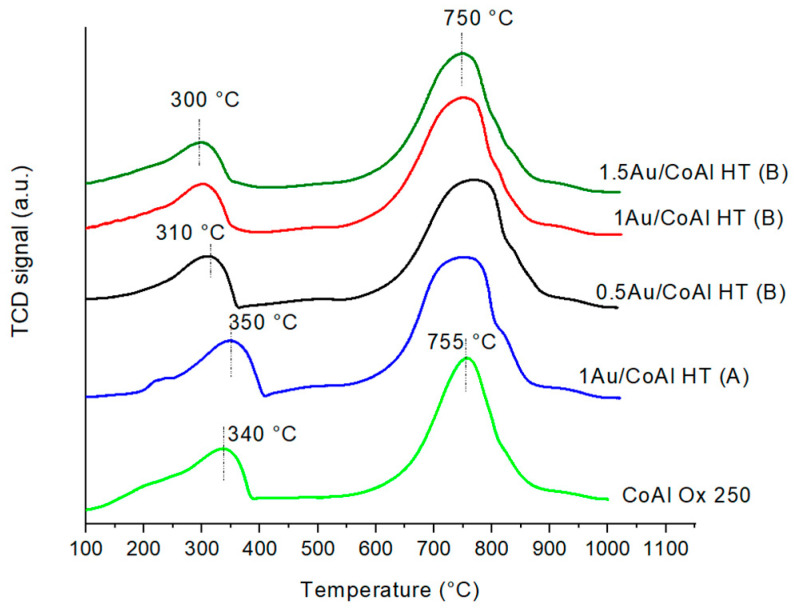
H_2_-TPR profiles for Au/Ox and Au/HT samples.

In order to understand the influence of method (B), the samples were characterized by H_2_-TPR. The H_2_-profiles are reported in Figure 7, where for comparison the reduction profile of the support CoAl Ox 250 has been added. The hydrogen consumption is reported in Table 2.

First of all, the reduction profile of all samples showed some similar behavior, with two reduction steps: the first one at low temperature (T < 400 °C), corresponding to a reduction in the Co_3_O_4_ phase to Co^0^, and the second one at higher temperature, with a reduction in Co-Al spinel phases (CoAl_2_O_4_ and Co_2_AlO_4_). This reduction is well documented in the literature [14,26,27,28,29,30]. Concerning the gold-based catalysts, it was observed that the 1Au/CoAl HT (A) showed the first reduction zone at higher temperature compared to the Au/CoAl HT (B) samples, while the presence of gold did not influence the position of the second peak, which remained centered at around 750 °C. The reduction in the active Co_3_O_4_ phase at lower temperature for Au catalysts prepared with method (B) can be explained by the strong interaction between Au nanoparticles and Co_3_O_4_ species [31,32,33]. This strong interaction was ascribed to the stabilization of [Au(OH)_4_]^−^ species in the layered structure of the LDH for catalysts with method (B) (as speculated by TGA, Figure 4), inducing a better dispersion of gold species compared to those with method (A). Looking at the hydrogen consumption (Table 2), it is worth noting that the 1Au/CoAl HT (A) exhibited the highest values for both peaks at both low and high temperature, whereas the hydrogen consumed by all the catalysts prepared with method (B) was the lowest. Such a trend suggests that in the latter samples Co was in a lower oxidation state, likely mainly Co^2+^, as confirmed by XPS analysis (see the Co^2+^/Co^3+^ atomic ratio).

In order to analyze the chemical composition and oxidation state of the surface species, XPS analysis of the prepared catalysts was performed.

**Figure 8:**
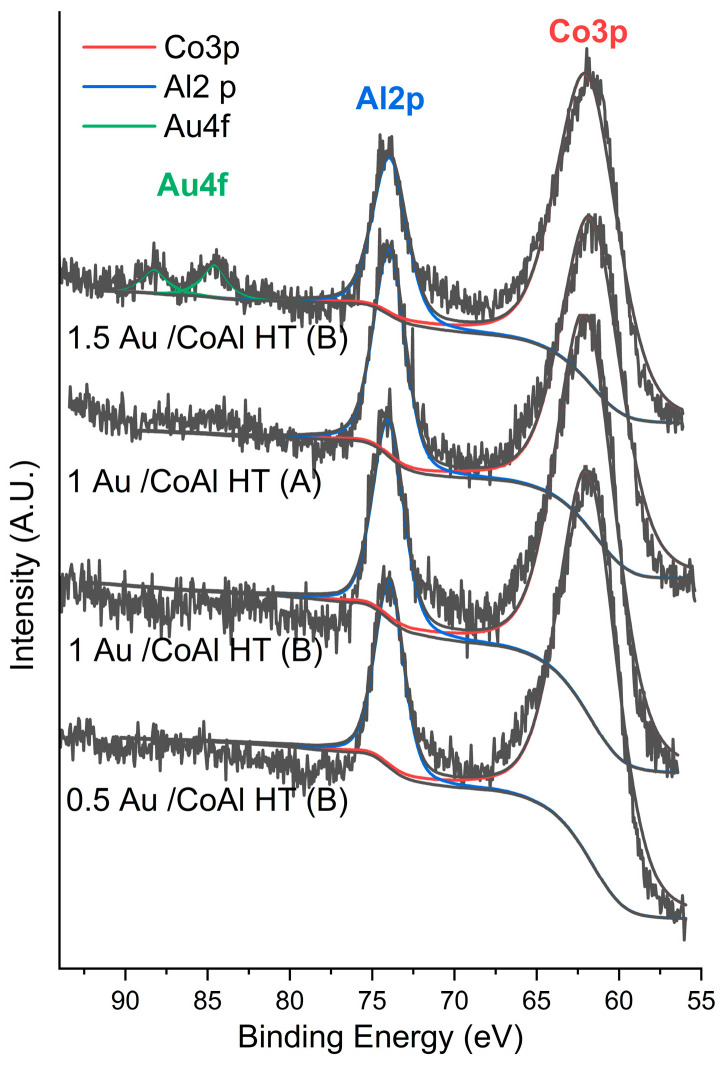
Co3p, Al2p, and Au4 XPS regions of CoAlOx and Au/CoAl HT samples.

The survey spectra confirmed the absence of contaminant elements such as K^+^ or Cl^−^. In order to evaluate the relative amount of cobalt, aluminum, and gold, the region between 55 and 94 eV was recorded. The region is shown in Figure 8 and the results are summarized in Table 2. In this region, reflections of Co 3p, Al2p, and Au4f were found, and the closeness of their binding energy allowed for a more precise evaluation of their relative amounts. As is found in analogous materials, the atomic ratio Co/Al ratio was lower than the bulk ratio, suggesting an aluminum surface enrichment [34,35]. For all samples, Al2p was centered at 73.9 and Co3p at 61.8 eV, which is compatible with Al (III) and Co (II) oxidation states [34]. Au4f peaks were not visible for 0.5Au/CoAl HT (B) or 1Au/CoAl HT (B), while in 1.5Au/CoAl HT (B), the amount of surface gold was enough to observe the two-spin orbit splitting peaks Au4f7/2 and Au4f5/2 with a separation of 3.6 eV. The binding energy of 83.9 eV is typical of metallic gold. The atomic ratio between Au4f7/2 and Al2p was 0.01. In the sample 1Au/CoAl HT (A), it was possible to see a faint presence of the two Au 4f peaks even though the intensity was not enough to allow for a proper analysis. Nevertheless, based on the comparison between 1Au/CoAl HT (B) and 1Au/CoAl HT (A), it appears that the latter material had a higher amount of gold on the surface.

**Figure 9:**
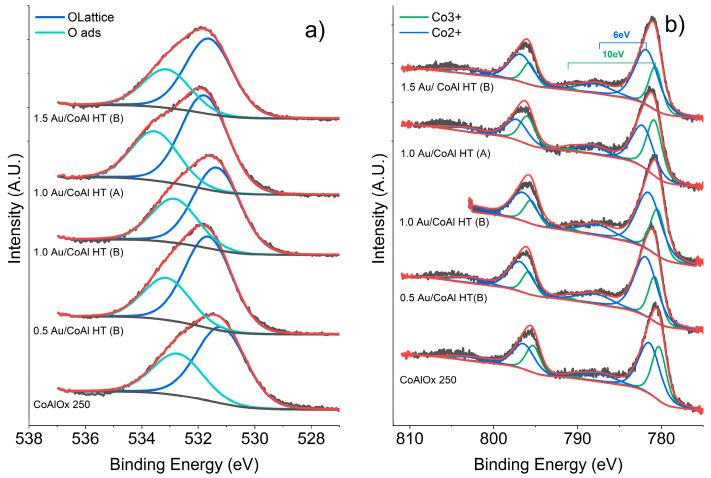
(**a**) O1s region and (**b**) Co2p region of CoAl Ox 250 and xAu/CoAl HT samples.

Figure 9a shows the profile and the deconvolution of the O1s region. For all samples, the peak was fitted with two components at 531 eV, attributed to oxygen from the lattice, and at 532.5 eV, attributed to surface adsorbed oxygen [36]. As expected, there was no variation between the relative amount of the two components, which for all samples was around 65% for the lattice component and 35% for the adsorbed species.

The Co2p region is presented in Figure 9b along with the deconvolution results. The two peaks, due to the Co2p3/2-Co2p1/2 spin orbit splitting, were further deconvoluted into two peaks attributed to Co(III) (780.8–795.8 eV) and Co(II) (781.9–796.8 eV). The presence of the two oxidation states was further confirmed by the presence of satellite peaks. Co(II) satellite is usually quite intense and with an energy gap of 6 eV with respect to the main Co2p peak, while the Co(III) satellite has an energy gap of 9–10 eV [37]. Taking into account all these constraints, the relative amount of Co(II) and Co(III) on the surface of the materials was calculated and the results are shown in Table 2. In all cases, Co(II) was the predominant species, with analogous ratios among the samples. The material prepared by method (A) showed a higher concentration of Co (III) on the surface, thus confirming the hypothesis of Co^2+^ oxidation into Co^3+^ during the synthesis with method (A) and consequent reduction of ionic Au species into metallic nanoparticles scarcely interacting with the support.

**Figure 10:**
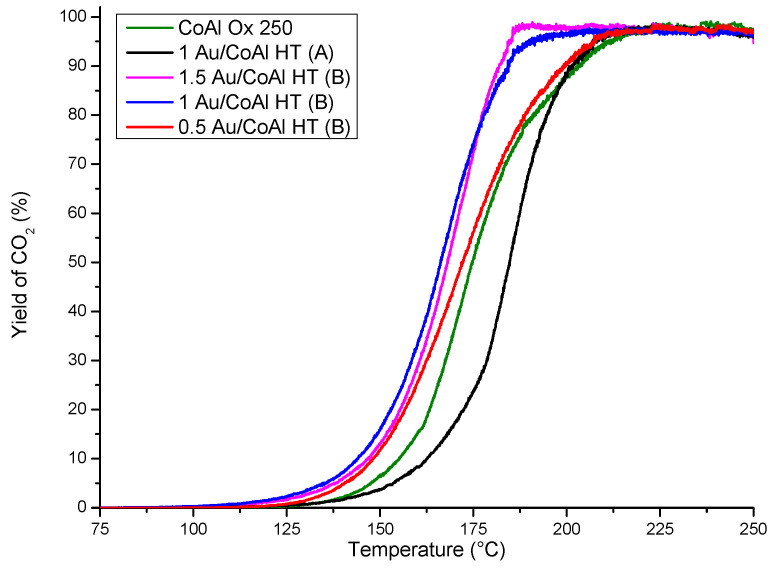
CO_2_ production during ethanol conversion vs. temperature.

The five gold-based samples were tested for the ethanol total oxidation. The production of carbon dioxide during the ethanol conversion curves is reported in Figure 10. The outlet gases after catalytic materials were analyzed by micro-gas chromatography and mass spectrometry. This metho of analysis allowed for the determination of the intermediate compounds before ethanol total oxidation. The principal by-products observed were acetaldehyde, methanol, ethylene, and, in lesser quantities, acetic acid, formic acid, diethyl ether, and ethyl acetate. The formation of acetaldehyde can either be formed from partial oxidation or by dehydrogenation at high temperatures of ethanol [26,27]. Traces of diethyl ether is formed by the dimerization of two ethanol molecules, while ethylene is produced by the dehydration of ethanol. Catalytic order based on T_50_ (temperature corresponding to 50% of conversion of ethanol into CO_2_) follows this order (Table 1):

1Au/CoAl HT (B) ≥ 1.5Au/CoAl HT (B) > 0.5Au/CoAl HT (B) > CoAl Ox 250 > 1Au/CoAl HT (A)

First of all, it was observed that the deposition of gold on the HT support by method (A) did not show interesting performance with a conversion at a higher temperature compared to the support. However, the samples prepared by method (B) showed interesting performance for the ethanol total oxidation and a positive influence from the presence of gold nanoparticles.

**Figure 11:**
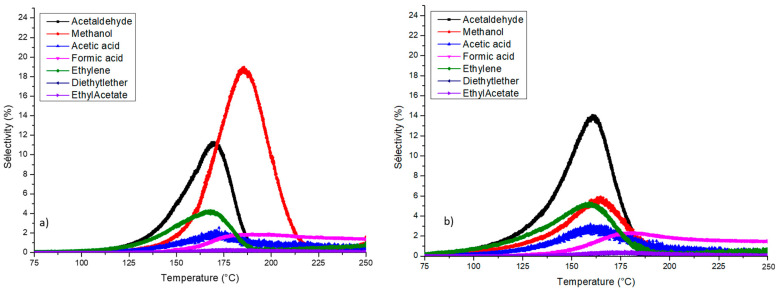
Evolution of by-product selectivity vs. temperature (**a**) CoAl Ox 250; (**b**) 1Au/CoAl HT (B).

The presence of gold nanoparticle also influenced the distribution of the byproducts formed during ethanol total oxidation. For this discussion, CoAl Ox 250 and 1Au/Col Al HT (B) samples were chosen as representative samples for the support and gold-based catalysts. The profiles of these by-products are represented in Figure 11 for the two samples.

At first view, the two profiles were quite different, in particular for the acetaldehyde and methanol profiles. Concerning the CoAl Ox, the main by-products observed were acetaldehyde and ethylene at low temperature, while methanol was formed at a higher temperature in significant concentrations (around 200 ppm). Concerning the gold samples, the behavior for the formation of by-products was similar regardless of the synthesis method of deposition. The quantity of acetaldehyde formed was similar to that of the CoAl Ox sample, while the selectivity for methanol was lower in case of gold-based materials. Concerning the influence of the gold content on samples synthetized by method (B), the maximum selectivity for the two mains products (acetaldehyde and methanol) are reported in the Table 1. Moreover, a difference in the temperature range for the formation of by-products was observed. Indeed, for the CoAl Ox, this temperature range was 110–225 °C, while for the 1AuHT (B) it was slightly lower, at 100–180 °C. This difference can be explained by the higher concentration of methanol formed for the CoAl Ox sample compared to the gold-based catalysts. This methanol can be oxidized more easily with gold nanoparticles compared to the CoAl Ox catalysts. To understand the reaction mechanism based on the by-products’ profiles, two reaction pathways are proposed. The first one concerns the partial oxidation of ethanol to acetaldehyde [28,38]. This acetaldehyde is converted to methanol before the oxidation to carbon dioxide and water molecules. Moreover, the total oxidation of acetaldehyde to carbon dioxide and water is also observed. The second pathway passes through the formation of ethylene before the formation of methanol or the total oxidation to CO_2_ and H_2_O [39].

**Figure 12:**
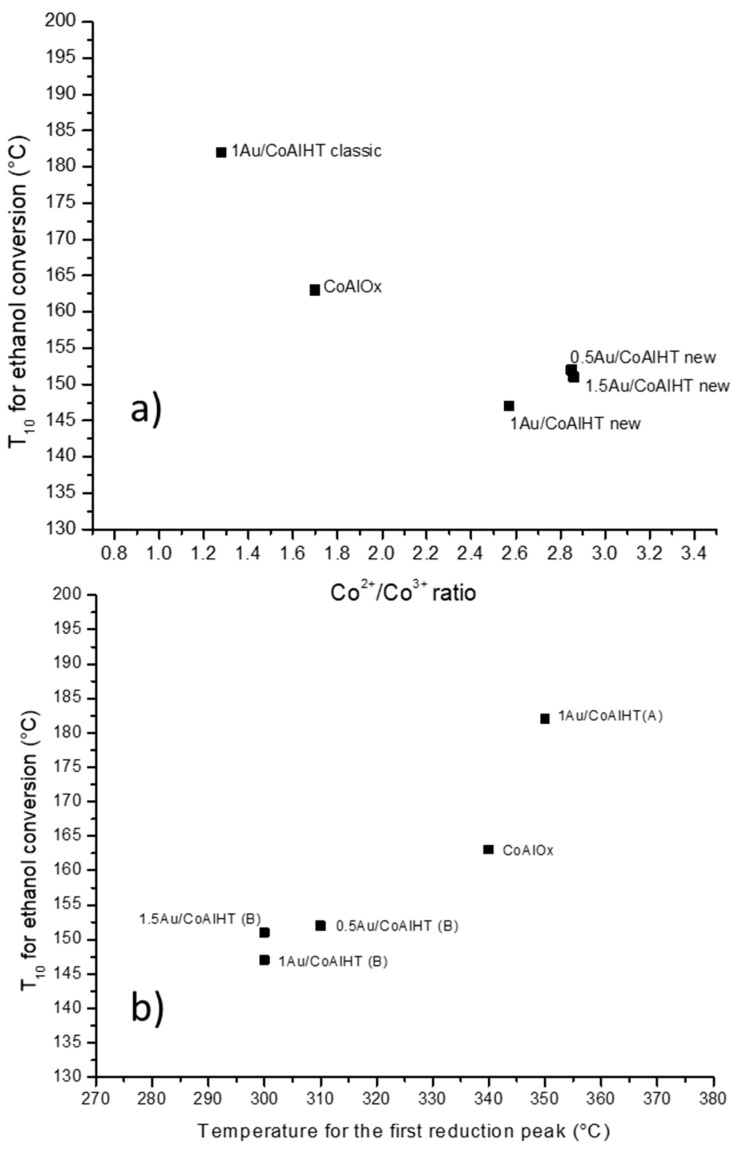
Relationship between T_10_ for ethanol conversion and Co^2+^/Co^3+^ surface ratio obtained by XPS analysis (**a**) and temperature of first reaction peak obtained by H_2_-TPR (**b**).

Based on the XPS and reducibility analyses, a clear trend can be observed. Indeed, a relationship between T_10_ obtained during the ethanol conversion into CO_2_ (see Table 1) and the Co^2+^/Co^3+^ ratio is plotted in Figure 12a, and a second relationship for T_10_ and the temperature of the first reduction peak by H_2_-TPR was also obtained (Figure 12b).

The linear trend reveals that the presence of surface Co^2+^ is a key factor for ethanol oxidation at low temperature, likely activating reactive oxygen species involved in the reaction [40]. Moreover, the ethanol oxidation activity at low temperature appeared to be well correlated to the temperature of the first reduction peak: the lower the T_10_, the lower the reduction temperature. These two relationships prove that the presence of surface oxygen vacancies, related to high concentration of Co^2+^, as well as high oxygen mobility at low temperature, induced by well-dispersed Au species strongly interacting with the support, are the key factors for ethanol catalytic conversion at low temperature.

Concerning the different selectivity to by-products over CoAl Ox 250 and 1Au/CoAl HT (B) (Figure 11), it can be proposed that acetaldehyde is principally oxidized by Co^2+^ species and gold nanoparticles into CO_2_ and water, whereas the CoAlOx with a larger proportion of Co^3+^ at the surface induces the formation of methanol at higher temperature.

## 3. Materials and Methods

### 3.1. Synthesis

Cobalt–aluminum-based layered double hydroxide (LDH) compounds are synthetized via the coprecipitation method [4,13]. The corresponding ratio M^2+^/M^3+^ used is equal to 3. A solution containing appropriate quantities of Co(NO_3_)_2_.3H_2_O and Al(NO_3_)_3_·9H_2_O is added dropwise, under vigorous stirring, to NaOH (2 M, ITW PanReac, Chicago, IL, USA, 98%) and Na_2_CO_3_ (1M, ACROS, Waltham, MA, USA, 99%) aqueous solution. The pH is maintained at 10.5 for 18 h at room temperature. The precipitate is then filtered, washed several times with hot deionized water (60 °C), and dried at 60 °C for 48 h. CoAl HT material is then obtained. Thermal treatments of CoAl HT at 250 °C (2 °C min^−1^) for 3 h and at 500 °C (1 °C min^−1^) for 4 h are performed to obtain oxide phase materials, denominated as CoAl Ox 250 and CoAl Ox 500, respectively.

Gold-supported nanoparticles are prepared using the deposition–precipitation (DP) method with two modifications. The first one, named (A), corresponds to the following methodology: In order to obtain 1 wt% of gold in the final catalyst, one gram of the support (CoAl HT, CoAl Ox 250, or CoAl Ox 500) is added, under stirring, to a proper volume of an aqueous solution of HAuCl_4_·3H_2_O (0.01 mol L^−1^; Aldrich, St. Louis, MO, USA, 99.999%). After 10 min, the pH of the HAuCl_4_ solution is adjusted to 8 by adding K_2_CO_3_ (0.05 mol L^−1^) drop by drop and stirring is maintained at r.t. for 3 h. It is worth noting that a darkening of the solution is observed as soon as the support is put in contact with the HAuCl_4_ solution, before adjusting the pH to 8. This behavior may be indicative of the reduction in gold and contextual oxidation of cobalt (Co^2+^ to Co^3+^) with the formation of gold particles scarcely interacting with the support. The so-formed suspension is heated at 65 °C under stirring overnight, and then the solid is filtered and washed several times with hot water (50 °C) in order to eliminate the K^+^ and Cl^−^ ions. The resulting catalyst is then dried in the oven at 80 °C followed by calcination at 250 °C (heating rate 2 °C min^−1^) for 3 h. The code names of these catalysts are 1Au/CoAl HT (A) for the use of hydrotalcite precursor as a support and 1Au/CoAl Ox 250 (A) or 1Au/CoAl Ox 500 (A) for the use of the mixed oxide calcined at 250 or at 500 °C, respectively.

The second method, named method (B), corresponds to the following: The pH of an appropriate volume of HAuCl_4_·3H_2_O (0.01 mol L^−1^; Aldrich, 99.999%) is fixed at 8 by adding K_2_CO_3_ (0.05 mol L^−1^; Aldrich, >99%), allowing for hydrolysis of the gold precursor before adding the support. At this step, the material powders CoAl HT or CoAl Ox 250 are added under stirring and the temperature is increased to 65 °C. The suspension is stirred overnight at this temperature. After filtering and careful washing with hot (50 °C) deionized water, the powder is dried overnight at 80 °C before being calcined at 250 °C for 3 h (heating rate 2 °C min^−1^). These samples are denoted as yAu/X (B), with y = 0.5, 1, or 1.5 wt%, expressing the Au weight percentage and X the corresponding support (CoAl HT or CoAl Ox 250).

### 3.2. Characterization

Differential thermal and thermogravimetric analyses (DTA/TG) (1 Star System, Mettler Toledo, Greifensee, Switzerland) are conducted in a stream (40 mL min^−1^) of air at a heating rate of 5 °C min^−1^ from room temperature to 625 °C with a CoAl HT sample mass of about 15 mg with the aim of evaluating the lowest temperature for the destruction of the LDH structure and the formation of the mixed oxide CoAl Ox. Another set of analyses is carried out over yAu/CoAl HT dried materials (y = 0.5, 1.0, 1.5 wt%) prepared by method (B), and the support CoAl HT is used for comparison. In order to simulate the calcination conditions, about 15 mg of sample are heated from r.t. to 250 °C at 2 °C min^−1^ under air flow (40 mL min^−1^).

The crystallinity of the supports and gold-based catalysts is analyzed at room temperature by X-ray diffraction analysis using a Bruker D8 Advance X-ray Diffractometer (AXS, Billerica, MA, USA) equipped with a CuKα radiation (λ = 1.5418 Å) and a Lynx Eye Detector. The measurements are performed from 5° to 80° with a step size Δ(2θ) = 0.02° and a counting time of 6 s per step. The crystalline phases are assigned by means of JCPDF cards. The Au mean particle size of selected catalysts is calculated by applying the Scherrer equation to the (111) peak (only if we report the mean particle size for the two Au catalysts by method (A): 1Au/CoAl Ox 500 and 1Au/CoAl Ox 250).

Specific surface area, pore volume, and pore size distribution of the samples are measured at −196 °C with a nitrogen sorption technique using ASAP 2020 equipment (Micromeritics, Norcross, GA, USA). Before the measurements, the powder samples (ca. 200 mg) are degassed at 250 °C for 2 h. The specific surface area is calculated via the Brunauer–Emmett–Teller (BET) method in the standard pressure range of 0.05–0.3 P/P_0_. The pore volume and pore size distribution are obtained by analysis of the desorption branch using the Barrett, Joyner, and Halenda (BJH) calculation method.

Temperature-programmed reduction (H_2_-TPR) experiments are carried out in a Micromeretics Autochem 2950 apparatus. Prior to the TPR experiment, a 100 mg sample is treated under argon at 150 °C for 1 h. The samples are then heated from ambient temperature to 1000 °C under H_2_ flow (5% vol. in argon—30 mL min^−1^), with a heating rate of 5 °C min^−1^.

The X-ray photoelectron spectroscopy (XPS) analyses are performed with a VG Microtech ESCA 3000Multilab (VG Scientific, Sussex, UK) equipped with a dual Mg/Al anode. Unmonochromatized Mg Kα radiation (1253.6 eV) is used as the excitation source. The sample powders are analyzed while mounted on double-sided adhesive tape. The pressure in the analysis chamber is in the range of 10^−8^ Torr during data collection. The constant charging of the samples is removed by referencing all the energies to the C1s at 285.0 eV. Analyses of the peaks are performed with the software CasaXPS version 2.3.26PR1.0. Atomic concentrations are calculated from peak intensity using the sensitivity factors provided by the software. The binding energy values are quoted with a precision of ±0.15 eV and the atomic percentage with a precision of ±10%.

Ethanol oxidation catalytic tests are carried out in a continuous-flow fixed-bed reactor loaded with 100 mg of catalyst at atmospheric pressure. The gas mixture composed of 1000 ppm ethanol/air passes through the reactor at a flow rate of 100 mL min^−1^, which corresponds to a gas hourly space velocity (GHSV) of about 30,000 h^−1^. Before each test, the catalysts are preactivated at 250 °C for 1 h under flowing air (33 mL min^−1^).

The reactants and the products of the oxidation of ethanol are analyzed using micro-gas chromatography (Varian CP-4900, Palo Alto, CA, USA) coupled to a Pfeiffer Vacuum Omnistar Quadrupole Mass Spectrometer (QMS-200, Aslar, Germany).

The ethanol conversion is calculated considering the CO_2_ formed during the reaction and in terms of the carbon number for each compound:

$$\mathrm{Where}:\;X_{T}=\frac{{\left[{CO}_{2}\right]}_{T}}{{2*[{C_{2}H}_{6}O]}_{0}}$$

where:

- X_T_ is the ethanol conversion at T temperature (%);
- [CO_2_]_T_ is the concentration of CO_2_ at T temperature (ppm).

The selectivity of the by-products is evaluated by taking into an account all the molecules formed at the temperature considered:

$$\mathrm{Where}:\;S_{I,T}=\frac{{n_{C}}_{I}\times {\left[I\right]}_{T}}{{2\times \left[{C_{2}H}_{6}O\right]}_{T}+{\left[{CH}_{4}O\right]}_{T}{+2\times \left[{C_{2}H}_{4}O_{2}\right]}_{T}{+2\times \left[{C_{2}H}_{4}\right]}_{T}+4{\times \left[{C_{4}H}_{10}O\right]}_{T}{+4\times \left[{C_{4}H}_{8}O_{2}\right]}_{T}{+\left[{CO}_{2}\right]}_{T}}$$

where:

- S_I_,_T_ is the selectivity of I at T temperature (%);
- ${n_{C}}_{I}$ is the number of C for compound I
- [I]_T_ is the concentration of I at T temperature (ppm).

## 4. Conclusions

Gold deposited on CoAl mixed oxide issued from layered double hydroxide (LDH) was synthetized and tested for the total oxidation of ethanol at low temperature. The deposition–precipitation (DP) method was applied with two modifications: related to the hydrolysis of the HAuCl_4_ precursor that was carried out after addition of the support to the solution, method (A), or before the addition of the support, method (B). Moreover, the two methods were applied using as a support the CoAl mixed oxide and the LDH precursor. Synthesis method (B) allowed the dispersion of gold nanoparticles over the LDH used as support to be enhanced and the oxidation state of cobalt as 2^+^ to be preserved, inducing better catalytic performance for ethanol oxidation at low temperature, with respect to the catalysts obtained with method (A). Conversely, the catalysts prepared by method (A) showed a higher concentration of Co (III) on the surface due to Co^2+^ oxidation into Co^3+^ during the synthesis and concomitant reduction in ionic Au species into metallic nanoparticles that scarcely interacted with the support. The presence of gold nanoparticles also induced an influence on the formed by-products, notably for methanol formation. Indeed, a lower concentration of methanol was observed in the presence of gold compared to the CoAlOx support.

## Figures and Tables

**Table 1:** Textural and catalytic properties of materials.

Samples	Specific Surface Area (m^2^·g^−1^)	Mean Pore Size (nm)	Pore Volume (cm^3^·g^−1^)	Ethanol Oxidation				
				T_10_ (°C)	T_50_ (°C)	Selectivity Max (%)		Carbon Balance (%)
						Methanol	Acetaldehyde	
CoAl Ox 250	275	16.7	0.95	155	176	11	18	96.7
CoAl Ox 500	113	20.0	0.67	174	196	10	17	97.2
1Au/CoAl Ox 250 (A)	155	11.5	0.53	165	188	7	13	97.5
1Au/CoAl Ox 500 (A)	108	15.6	0.55	165	190	6	14	96.8
1Au/CoAl HT (A)	180	12.0	0.52	159	184	7	13	96.5
0.5Au/CoAl HT (B)	224	10.9	0.60	148	171	3	9	98.0
1Au/CoAl HT (B)	206	11.2	0.57	143	166	7	14	98.9
1.5Au/CoAl HT (B)	197	11.1	0.56	146	168	5	16	98.4

**Table 2:** H_2_ consumption and XPS atomic ratio for the gold-based catalysts.

Samples	H_2_ Consumption (mL/g)			XPS Atomic Ratio		
	Low Temperature (<400 °C)	High Temperature (>400 °C)	Total	Co^2+^/Co^3+^	Co/Al	Au/Al
CoAl Ox 250	69	215	284	1.70	1.05	-
1Au/CoAl HT (A)	72	221	293	1.28	0.97	n.d. *
0.5Au/CoAl HT (B)	63	200	263	2.85	1.19	n.d.
1Au/CoAl HT (B)	63	201	264	2.57	1.07	n.d.
1.5Au/CoAl HT (B)	66	195	261	2.86	1.08	0.01

* n.d. gold signal not detected.

## Data Availability

Data are contained within the article.
